# The Influence of Family Support According to Gender in the Portuguese Language Course Achievement

**DOI:** 10.3389/fpsyg.2017.01610

**Published:** 2017-09-19

**Authors:** Heldemerina S. Pires, Adelinda A. Candeias, Luísa Grácio, Edgar Galindo, Madalena Melo

**Affiliations:** ^1^Department of Psychology, School of Social Sciences, University of Évora Évora, Portugal; ^2^Center for Research in Education and Psychology, University of Évora Évora, Portugal; ^3^Interdisciplinary Center for History, Culture and Societies, University of Évora Évora, Portugal

**Keywords:** achievement, Portuguese language course, family support, gender differences, attitudes

## Abstract

Several factors like pupils’ characteristics, school conditions and family context have been pointed out in the literature as being linked to academic achievement. Regarding the latter, family socio-economic status and parental support have been identified as determining variables on success at school. The current study analyses the influence of family support on the achievement of school children in their native language [Portuguese language course (PLC)]. Participants were 885 students attending PLC on basic and secondary schools (6th and 9th school grades) (ISCED 1); 418 boys and 467 girls, ranged between 10 and 18 years of age (*M =* 12.99). School achievement was assessed using year-end classifications in PLC. Family support was assessed using the Family Support-Context Variables Questionnaire. A regression analysis showed that students’ perception about instrumental and affective family support in school life was positively related to their Portuguese grades. However, different predictive values were revealed according to gender. Girls’ Portuguese languge couse classification seemed to depend on affective variables like their perception of affective parental support and affective attitudes toward the PLC, while boys’ results seemed to be influenced by instrumental variables, such as the perception of instrumental support from parents and boys’ attitudes toward the utility of learning Portuguese language. These results supported those of other studies, showing the need to take gender differences into account in educational interventions, especially during early adolescence. In conclusion, the study shows an influence of parental support on PLC achievement. Such influence differs according to gender, with girls being more sensitive to the affective dimension of parental support and boys to the instrumental one.

## Introduction

Children’s school achievement may be influenced by a variety of individual and environmental factors (Kerr, 2014). Several authors point to the fact that academic achievement is determined by students’ personal factors, family aspects and characteristics of the school (González-Pienda et al., 2002; Jeynes, 2007; Blondal and Adalbjarnardottir, 2009; Fan and Williams, 2010; Karibayeva and Bogar, 2014; Kerr, 2014). The current view of school achievement and failure emphasizes the role of the environment as a determining factor in children’s achievement. Consequently, academic success has been studied not only from the point of view of individual characteristics, but also as a function of environmental variables, according to the ecological model of Bronfenbrenner (1979) that points to reciprocal influences among the various systems, namely family and school, in our case.

School achievement has been analyzed as a function of individual variables, like gender (King, 2016), academic self-concept (e.g., Chen et al., 2013), pupils’ perception of parental attitudes (e.g., Peixoto and Carvalho, 2009), children’s emotions (e.g., Valiente et al., 2012), and diverse motivational (e.g., Wigfield et al., 2015) and cognitive (Dent and Koenka, 2016) dimensions.

In respect to environmental factors, relevant studies have analyzed the relationships between academic achievement and the school system, including variables like teachers’ attitudes toward gender and race (Zusho et al., 2016), relationships between teachers (Dodge et al., 2003; Gauvain, 2016; Hanish et al., 2016), and pupil-teacher relationships (Montague and Rinaldi, 2001; Gregory and Korth, 2016; Wubbels et al., 2016). Other authors, such as Brown (2009), have analyzed the relevance of more general factors like social economic status/SES for school achievement. Finally, a set of studies has analyzed the relevance of family characteristics for school achievement and failure (Kurdek and Sinclair, 2001; Abbott, 2012; Karibayeva and Bogar, 2014; Castro et al., 2015), particularly parental support (Baker et al., 2001; Dearing et al., 2004; Goodall and Montgomery, 2014; Bazán and Castellanos, 2015; You et al., 2016). The current study focuses precisely on this latter factor, namely, the influence of family support on achievement in a specific subject, Portuguese as a native language.

At an individual level, the mastery of language is considered key to general learning and specifically to the learning of a range of school subjects. It has been seen as a pre-condition for learning in general and, especially, for reading, writing and maths (Poe et al., 2004). Language problems can have immediate negative consequences for children’s progress at school and may hinder the subsequent acquisition of important adult life skills and integration into society (European Parliament, 2006). On the other hand, the native language is also a part of individuals’ personal identity and a direct cultural expression (Mateus, 2011). In the school setting, language learning (Portuguese language, in the current study) has a central role in the curriculum. It is the basis for learning the content of other school subjects, as it serves as a transversal skill for all disciplines (Costa et al., 2000). The mastery of one’s native language is considered a general skill for the development of the individual in society (Valadares, 2003; Figel, 2007; Organisation for Economic Co-operation, and Development [OECD], 2012; Organisation for Economic Co-operation and Development [OECD], 2014).

The family has been identified as a major environmental influence (Holloway and Jonas, 2016), determining school achievement of children and adolescents (Claes et al., 2003; Abbott, 2012; Bazán and Castellanos, 2015). Some studies show that parents involved in school positively influence the achievement of their children, i.e., the pupils show more commitment to school tasks and their dropout levels are lower (Jeynes, 2007; Park and Holloway, 2013). Also, children and adolescents living in a supportive family context normally have more parental help in school tasks (Alves-Martins and Peixoto, 2000; Alves-Martins et al., 2002). In general terms, good academic achievement is associated with respectful, stable, balanced families (Alvarenga and Piccinini, 2002). Parental affective and material support increases both school adjustment and the motivation of students and leads to better levels of school achievement (McGrath and Repetti, 2000; Ryan and Patrick, 2001).

According to Chowa et al. (2013), parental support has mediating effects on the attitudes of children toward school. Moreover, it fosters adaptive academic practices and transmits positive information about school. However, the effect of parental involvement depends on its type. Parental involvement at home (e.g., homework support, talking to their child about what they learned in school) is positively associated with children’s achievement in the native language (English, in this case) and maths (with a smaller size effect). On the other hand, parental involvement in school (e.g., parents’ attendance at meetings with teachers, participation in school events) is negatively associated with school achievement, namely in maths and English classifications (Chowa et al., 2013). The authors suggest that such negative relationship may be due to the fact that greater involvement of parents can be a result of being called to school due to pupil’s problems (children’s low achievement leads to an increase in parental involvement). However, Wilder (2014), based on a meta-synthesis of meta-analyses on the effects of parental involvement in academic achievement, finds a positive relationship between involvement (regardless of their definition) and academic outcomes.

The main goal of the current study is to analyze the influence of parental support on the achievement of school children in a specific subject, Portuguese language course (PLC) native language. Year-end classifications in PLC were also looked in relation to the type of parental support (affective or instrumental), student attitudes (motivation, affect, and utility) toward PLC and, finally, the students’ age and gender.

## Materials and Methods

### Participants

Participants were 885 students attending 6th and 9th school grades, International Standard Classification of Education (ISCED 1) in four Portuguese regions (23.8% in the north; 19.7% in the center; 18.0% in Lisbon region; 17.4% in the Alentejo; 13.6% Algarve; and 7.3% Azores). 467 (52.8%) were girls and 418 (47.2%) boys, and they ranged between 10 and 18 years of age (*M =* 12.99; *SD* = 1.63).

### Instruments

The *Questionnaire* of Contextual variables – Family Support (QCV-FS; Pires et al., 2013) comprised 13 items measuring parental support. The format was a four-category Likert-type ranging from *strongly agree* (four points) to *strongly disagree* (one point). The QCV-FS items are organized into two dimensions:

(i) *Affective support* (seven items) – e.g., *My family helps me to feel better when I am worried.*
(ii) *Instrumental support* (six items) – e.g., *My family goes over my school results with me.*

These two dimensions account for 57.8% of the total variance. The internal consistency analyzed by Alfa Cronbach measured 0.911 for the global tool, 0.884 for affective support, and 0.816 for the instrumental.

The *Attitude toward Portuguese Language Course Questionnaire* (ATPCQ, Neto et al., 2011) comprised 17 items, each answered in a four-category Likert-type response format ranging from *strongly agree* (four point) to *strongly disagree* (one point). The negatively worded items were reversely scored, leading to the self-perception and appreciation of the student as a person and as a student. These items are organized into three dimensions:

(i) *Motivation* (four items) – e.g., *I feel enthusiastic when I go to Portuguese language classes.*
(ii) *Affection* (seven items) – e.g., *The discipline of Portuguese makes me feel insecure.*
(iii) *Utility* (six items) – e.g., *The Portuguese language is useful for my life.*

The items from the second dimension were negative, so we proceeded to recodify them so that they appear to be positive in the data analysis and could be analyzed as positive feelings about learning the Portuguese language.

These three dimensions explained 60% of the variance, with a Cronbach Alpha of 0.90 (Neto et al., 2011).

Portuguese language course achievement was measured by the year-end school classifications in a quantitative scale from 1 to 5. The classifications at 6th and 9th school grades are comparable because it is a unique measurement scale and each value has the same meaning (1 is a bad classification; 5 a very good classification). Year-end classifications are the weighted result of the school internal classification and the classification of national examinations. In the case of Portugal, it is important to mention that there is a national curriculum for every school year that is followed by all schools. The school grades were selected to measure achievement because they include information about school classification over a long period of time, and based on different sources of data, as participation in classes or written assessment, as some authors defend (Lemos et al., 2008; Roth et al., 2015).

### Procedure

This study is part of a larger project entitled: “School Income and Development” (RED). Firstly, we collected the authorizations to carry out the study from the Portuguese Commission for Data Protection, the Committee for Monitoring Surveys in Schools from the Portuguese Ministry of Education, and the principals of all participating schools. Students’ parents and the students themselves gave authorization as well. After the authorizations were granted, we applied the QCV-FS and the ATPCQ in a single, 40-min session during class time. In each session, the project was presented and all ethical and confidentiality conditions were carried out to safeguard data collection.

### Data Analysis

All the statistical procedures and tests were conducted using the IBM SPSS Statistics for Windows (version 21). A descriptive analysis of the sample’s characteristics was carried out. The missing values in QCV-FS and ATPCQ were replaced by the mean value obtained for all the participants with valid answers (Cuesta et al., 2013).

In our study there are no missing data on the variable “Classification in Portuguese Language Course Classifications,” but we accept cases with a maximum of three missing data in the questionnaires: QCV-FS and ATPCQ because in this questionnaire the participants had a list of items to answer. Those items are integrated in a factorial dimension with content convergence. So, we decided to accept the cases with one, two or three missing data and replace exactly these missing data by the mean value of the item obtained for all the participants with valid answers. We chose that procedure of replacing the missing data based in authors such as Peugh and Enders (2004) and Cuesta et al. (2013) because they show that this procedure is more precise and maintains the factorial structure of the questionnaires.

## Results

### Correlations among Variables and Rationale for Regression Models

Correlations among the study variables are given in **Table 1**. The results showed positive and significant correlations (*p* < 0.01), between the perception of family support – affective (*r* = 0.137) and family support – instrumental (*r* = 0.173) and the PLC classifications as well as between (*r* = 0.123), Attitude – affective (*r* = 0.134) and Attitude – utility (*r* = 0.173) and PLC classifications.

**Table 1:** Descriptive statistics and Zero-order correlation among study variables.

Variable	Mean (*SD*)	1	2	3	4	5	6	7
(1) Student age	12.99 (0.06)	–	-0.149^∗∗^	-0.406^∗∗^	0.075^∗^	-0.191^∗∗^	-0.206^∗∗^	-0.125^∗∗^
(2) Classifications in PLC	3.23 (0.03)		–	0.123^∗∗^	0.134^∗∗^	0.173^∗∗^	0.137^∗∗^	0.173^∗∗^
(3) Attitude – motivation	1.88 (0.02)			–	0.159^∗∗^	0.542^∗∗^	0.287^∗∗^	0.229^∗∗^
(4) Attitude – affective	2.16 (0.02)				–	0.341^∗∗^	0.042	0.143^∗∗^
(5) Attitude – utility	2.52 (0.02)					–	0.272^∗∗^	0.331^∗∗^
(6) Family support affective	2.27 (0.01)						–	0.626^∗∗^
(7) Family support instrumental	2.24 (0.01)							–

*N = (885); *^∗^p <* 0.05; *^∗∗^p* < 0.01.*

Parental support – Affective (*r* = -0.206) and instrumental (*r* = -0.125) were negatively correlated (*p* < 0.01) with students’ age. Classifications at PLC (*r* = -0.149), the attitudes – motivation (*r* = -0.406) and utility (*r* = -0.191) also correlated negatively with age. Thus, as students got older, not only did their academic achievement in PLC diminish, but there was also a fall in both their perception of affective and instrumental parental support as well as in their motivational and instrumental attitudes toward the language. On the other hand, perceptions of family support correlated positively, if weakly, with PLC and the latter with the attitudes toward the language.

The perception of family support – affective, was strongly and positively related to the perception of family support – instrumental (*r* = 0.626), that is, when the students felt affectively supported, they also perceived instrumental support from the parents. On the other hand, attitudes toward the PLC in the motivational, affective and instrumental (utility) dimensions correlated positively and significantly (*p* < 0.01). The motivational attitudes were related not only to the affective attitudes (*r* = 0.159) but also with the instrumental ones (*r* = 0.542); the affective and instrumental attitudes also correlated with each other (*r* = 0.341).

The perception of family support-affective correlated positively with motivational (*r* = 0.287) and instrumental (*r* = 0.272) attitudes toward the PLC. As far as the perception of instrumental support was concerned, there was also a positive correlation with all attitudinal dimensions: motivation (*r* = 0.229), affect (*r* = 0.143), and utility (*r* = 0.331).

There were statistically significant differences (*p* < 0.001) between boys and girls in their classifications in the PLC. Girls also demonstrated higher levels of positive attitudes toward PLC than boys in both the affective (*t* = 6.39); (*t* = 4.65) and instrumental dimensions (*t* = 3.92).

These results led us to examine the predictive power of perceptions of family support, and of attitudinal and developmental variables (age) relative to PLC classifications. Thus, we tested a global model to evaluate the predictive power of these variables on students’ PLC classifications (**Table 2**). Given the mean differences according to gender, different regression models were also tested for boys and girls (**Tables 3, 4**). These models were intended to verify if there were different explanatory patterns for boys and girls in the perception of family support, attitudes, and age in predicting PLC classifications.

**Table 2:** Summary of stepwise multiple regression analysis predicting academic classification in PLC.

Model	*B*	*SE*	β	*t*	Significance
Intercept	3.546	0.326		10.889	0.000
Gender	-0.314	0.052	-0.195	-6.005	0.000
Family support – instrumental	0.340	0.086	0.129	3.962	0.000
Age	-0.074	0.016	-0.149	**-**4.592	0.000
Attitude – affective	0.159	0.054	0.097	2.956	0.003

*r^*2*^ = 0.099; *r*^*2*^ = 0.095; *F*(4.880) = 24.066; *p* < 0.001.*

**Table 3:** Summary of stepwise multiple regression analysis predicting girls’ PLC classifications.

Model	*B*	*SE*	β	*t*	Significance
Intercept	3.460	0.441		7.846	0.000
Age	-0.080	0.024	-0.158	-3.388	0.001
Attitude – affective	0.201	0.081	0.114	2.484	0.013
Family support –affective	0.233	0.106	0.101	2.188	0.029

*r^*2*^= 0.047; *r*^*2*^= 0.041; *F*(3.466) = 7.642; *p* < 0.001.*

**Table 4:** Summary of stepwise multiple regression analysis predicting boys’ PLC classifications.

Model	*B*	*SE*	β	*t*	Significance
Intercept	2.340	0.462		5.063	0.000
Family support – instrumental	0.385	0.129	0.155	2.979	0.003
Attitude – utility	0.180	0.079	0.122	2.285	0.023
Age	-0.045	0.023	-0.099	-1.980	0.048

*r^*2*^= 0.078; *r*^*2*^= 0.071; *F*(3.414) = 11.673; *p* < 0.001.*

As can be seen (**Table 2**), in the general model (with a global predictive value of 9.5%), gender is the most important predictive variable (4.3%), followed by the perception of family support-instrumental (2.5%), age (1.9%) and attitudinal dimension- affective (0.8%).

There were also gender differences in the predictor variables (**Tables 3, 4**).

Thus, in girls, the variables with the greatest impact on classification in PLC (with a global predictive value of 4.1%) were: age (2.2%), affective attitudes (1.1%) and perception of parental affective support (0.8%).

For boys, the predictive variables with the greatest impact (with a global predictive value of 7.1%) were instrumental parental support (4.9%), instrumental attitudes toward PLC (1.6%) and age (0.6%).

## Discussion

The current study analyzes the influence of parental support on the achievement of school children in a specific subject: Portuguese as a native language. The relationships between the year-end Portuguese grade, the type of parental support (affective or instrumental), students’ attitudes (motivation, affect, and utility) toward the subject and the age and gender of students were also analyzed. Results showed that perceptions of parental support, age and favorable student’s attitudes correlated positively with classifications in PLC. Significant positive correlations (*p* < 0.01) were found between the perception of instrumental and affective parental support and classifications in PLC, as well as between the three dimensions of attitudes (motivation, affect, and utility) and language performance. **Table 1** shows that perception of affective parental support correlated positively with motivational and utility attitudes to the PLC as well as with marks in Portuguese language. On the other hand, perception of instrumental parental support correlated positively with the three dimensions of attitudes (utility, affect, and motivation). These results point to the existence of reciprocal influences among those variables, where parental support seems to be a variable with multidimensional influence and that has an important impact on PLC classifications and consequently on achievement. These findings support those of other researchers (Malecki and Demaray, 2003; Ratelle et al., 2005; Chowa et al., 2013; Karbach et al., 2013; Wilder, 2014; Castro et al., 2015; Song et al., 2015; King, 2016; Arens and Jude, 2017).

The current results also supported those of other studies (e.g., Martin and Steinbeck, 2017) showing that motivation played an important role in performance, since low motivation is associated with low achievement and high motivation to high performance. The variable affect also demonstrated a previously identified (see Williams et al., 2005; Rogaten et al., 2013), narrow link with motivation and performance. Positive emotions were seen to support academic achievement, when mediated by self-regulated learning and motivation (Mega et al., 2014). It must be pointed out, however, that, in spite of finding positive correlations, the results also revealed a parallel decrease of motivation and PLC classifications with increasing student’s age. These results coincided with those of Mucherah and Ambrose-Stahl (2014) and Martin and Steinbeck (2017) who found a decrease in motivational levels of students in puberty, as did Bozack (2011), who mentioned teachers’ difficulty in motivating students at that age. In the current study, the motivational decrease is associated with a fall in performance, a fact which corresponds with Martin and Steinbeck (2017) results, pointing to a mutual influence between the variables.

It is verified that affective and instrumental parental support has a significant negative correlation (*p* < 0.01) with age. The significant negative correlation between the perception of parental support and age means that, as age increases, a decline in the amount of parental support is perceived. At this stage of development, adolescents generally experience increasing autonomy from their parents in favor of greater peer-to-peer communication/attachment, a fact which may be influencing the perception of decreased parental support (Song et al., 2015). Nevertheless, in our findings, parental support is (albeit slightly) associated with good classifications in PLC, even if students report falling support. This fact seems to demonstrate, once again, the importance of parental support.

An analysis of age pointed out a significantly negative correlation (*p* < 0.01) between PLC classifications and the motivational and utilitarian attitudes toward the language. There were also significant differences in language grades by gender, with girls having higher means. This has been confirmed by other studies (e.g., King, 2016) that showed better achievement for girls.

In the current study, the gender difference became more noticeable when examining its predictive power. These results allowed us to confirm the existence of different predictive patterns in relation to PLC classifications according to gender. Thus, for girls, academic achievement in PLC was predicted by age, affective attitudes and the perception of parental affective support. In boys, the predictable variables were, in order of importance, perceptions of parental instrumental support, instrumental attitudes toward the PLC and, finally, age. These results clearly supported the findings that language classifications were influenced by variables of a different nature in boys and girls. On the one hand, girls appeared to be more susceptible to the influence of affective variables (perception of parental affective support and affective attitudes). On the other, boys appeared to be more sensitive to the influence of instrumental variables (perception of parental instrumental support and instrumental attitudes). The students’ age for both genders served as a negative predictor of classification in PLC. This result refers to the influence of developmental variables on academic achievement and has been supported by other research (e.g., Martin and Steinbeck, 2017) showing a link between puberty (developmental variable) and decreasing academic achievement.

It is important to highlight that, for boys, instrumentality has special importance in the perception of parental support, as well as in attitudes. Parental instrumental support (e.g., encouragement, appreciation of value, discussion about the marks) is recognized and valued, influencing boys’ achievement in PLC and it is also linked to the perception of the subject’s utility. There was some consistency in these results because, in some situations, instrumental support was driven by other reasons (Morelli et al., 2015). For girls, the affective dimension was of special importance. PLC classifications was mainly associated with the affective components of parental support (e.g., encouragement, respect, understanding) of the attitudinal dimensions (e.g., insecurity, unpleasant feeling). Studies (e.g., Morelli et al., 2015) suggest that, in some cases, emotional support and instrumental support are linked. Nevertheless, there are also differences between the two types of support in individuals. In the present study, this link was demonstrated by a positive correlation between the perceptions of parental affective and instrumental support. This means that when students felt affectionately supported, they also perceived instrumental support from their parents (*r* = 0.626). According to Ratelle et al. (2005), perceptions of parental involvement and emotional support play a specific role in predicting the student’s performance. Furthermore, these perceptions are especially correlated with language performance, particularly regarding the native language. In agreement with Porumbu and Necşoi (2013), there was a consistent link between the student’s academic achievement and variables associated with parents’ support of their children’s school life. Khajehpoura and Ghazvini (2011) also demonstrated that when parents are encouraged to support their children’s school life, children’s grades definitely improve. According to Porumbu and Necşoi (2013), these data suggest that schools have to review their practices of involving parents in the communication between school and family. Moreover, parents should be aided in developing the necessary skills for supporting their children. This need is reinforced by the evidence of the differential effects of parental support on children’s results (Chowa et al., 2013; Karbach et al., 2013).

## Conclusion

The current results allow us to conclude that parental support influences PLC achievement. This influence is differentiated according to gender, with girls being more sensitive to the affective dimension of parental support and boys to the instrumental one.

In general, these results have some practical implications related to the implementation and use of differentiated strategies in the fight against school failure. It is suggested that strategies must be differentiated at the level of educational practice. Several suggestions follow: (i) In the family context, parental practical support should be differentiated by gender: instrumental parental support in boys and affective support in girls. However, it is important to point out that there is an influence between the affective and instrumental supports, since in some situations the instrumental support is accompanied by affective support and both establish a relationship with effects on well-being (Arens and Jude, 2017) Thus, the affective support provided by instrumental support can generate positive effects that, in addition to improvements in performance, may have implications for the students’ well-being and mental health. (ii) Communication and connection between the school and the family should promote and encourage parents to monitor children’s school life. (iii) Age (relative to puberty) is a developmental variable with negative influence on motivation both in boys and in girls and both (pre/post puberty and motivation) are predictors of academic achievement (Martin and Steinbeck, 2017). So, since motivational difficulties are more present in older, post-puberty students (Martin and Steinbeck, 2017), it is important that strategies for improving motivation and school achievement also take this factor into consideration.

## Author Contributions

Conceptualization: HP and AC; methodology: AC; formal analysis: AC and MM; investigation: HP and LG; wrote the manuscript: HP, MM, LG, and AC; reviewed, and edited the manuscript: EG, LG, and MM; supervision: EG.

## Conflict of Interest Statement

The authors declare that the research was conducted in the absence of any commercial or financial relationships that could be construed as a potential conflict of interest.
